# Refining a capability development framework for building successful consumer and staff partnerships in healthcare quality improvement: A coproduced eDelphi study

**DOI:** 10.1111/hex.13499

**Published:** 2022-04-26

**Authors:** Ruth Cox, Melissa Kendall, Matthew Molineux, Elizabeth Miller, Bernadette Tanner

**Affiliations:** ^1^ Occupational Therapy Department Queen Elizabeth II Jubilee Hospital Coopers Plains Queensland Australia; ^2^ Discipline of Occupational Therapy, School of Health Sciences and Social Work Griffith University Queensland Australia; ^3^ Acquired Brain Injury Outreach Service and Transitional Rehabilitation Program Princess Alexandra Hospital Buranda Queensland Australia; ^4^ School of Health Sciences and Social Work Griffith University Queensland Australia; ^5^ Queen Elizabeth II Jubilee Hospital Coopers Plains Queensland Australia

**Keywords:** capability, consumer and community involvement, Delphi, learning, partnerships, quality improvement, training

## Abstract

**Background:**

The capability of consumers and staff may be critical for authentic and effective partnerships in healthcare quality improvement (QI). Capability frameworks describe core knowledge, skills, values, attitudes, and behaviours and guide learning and development at individual and organizational levels.

**Objective:**

To refine a capability framework for successful partnerships in healthcare QI which was coproduced from a scoping review.

**Design:**

A two‐round eDelphi design was used. The International Expert Panel rated the importance of framework items in supporting successful QI partnerships, and suggested improvements. They also rated implementation options and commented on the influence of context.

**Participants:**

Seven Research Advisory Group members were recruited to support the research team. The eDelphi panel included 53 people, with 44 (83%) and 42 (77. 8%) participating in rounds 1 and 2, respectively. They were from eight countries and had diverse backgrounds.

**Results:**

The Research Advisory Group and panel endorsed the framework and summary diagram as valuable resources to support the growth of authentic and meaningful partnerships in QI across healthcare contexts, conditions, and countries. A consensus was established on content and structure. Substantial rewording included a stronger emphasis on growth, trust, respect, inclusivity, diversity, and challenging the status quo. The final capability development framework included three domains: *Personal Attributes*, *Relationships and Communication*, and *Principles and Practices*. The *Equalizing Decision Making, Power, and Leadership* capability was foundational and positioned across all domains. Ten capabilities with twenty‐seven capability descriptions were also included. The *Principles and Practices* domain, *Equalizing Decision Making, Power, and Leadership* capability, and almost half (44.4%) of the capability descriptions were rated as more important for staff than consumers (*p* < .01). However, only the QI processes and practices capability description did not meet the inclusion threshold for consumers. Thus, the framework was applicable to staff and consumers.

**Conclusion:**

The refined capability development framework provides direction for planning and provision of learning and development regarding QI partnerships.

**Patient or Public Contribution:**

Two consumers were full members of the research team and are coauthors. A Research Advisory Group, inclusive of consumers, guided study execution and translation planning. More than half of the panel were consumers.

## 1 INTRODUCTION

Globally, patient and family engagement is gaining momentum as a strategy to improve quality and safety across the continuum of health services.^1^, ^2^ The language used internationally to encompass the concepts of patient and family engagement varies and includes patient and public participation, patient and public involvement and engagement, stakeholder engagement, and consumer and community involvement (CCI). CCI will be used throughout this paper as the research originated in Australia where that terminology is widely used. The term consumer is inclusive of past, present, and future health service users (patients), family members, and the public or community.^3^ Including consumer partnerships in service planning, delivery, and evaluation aims to improve person‐centred care and is linked with the paradigms of consumerism, democracy, human rights, recovery, and empowerment.^4^ A systematic review reported benefits from CCI in healthcare quality improvement (QI) including improved service delivery, enhanced governance, and better‐informed policies and planning.^5^ Despite these benefits, authentic partnerships in QI are not the norm and changes are needed at the individual, organizational, and system levels.^6^, ^7^, ^8^ In particular, both individual consumers and healthcare staff need the requisite attitudes, skills, and knowledge to successfully partner in QI.^9^

The provision of education for partnership capabilities has been recognized as an enabler of effective CCI with health professionals noting that this is not taught in their health degrees.^6^ Furthermore, the World Health Organization has recommended that ongoing learning for effective involvement of patients in quality and safety should be a requirement for professional registration.^1^ Additionally, training for both consumers and staff is included in policy and accreditation requirements in many health jurisdictions,^3^, ^10^, ^11^ and research has also highlighted the need for training and development.^6^, ^12^, ^13^, ^14^, ^15^ However, despite these calls for staff and consumer education, little research provides clear direction for the planning and provision of learning initiatives to enhance partnership capabilities. Capability frameworks may be an effective strategy to address this need as they guide learning and development planning and implementation at the individual and organizational level by describing core knowledge, skills, values, attitudes, and behaviours.^16^, ^17^, ^18^, ^19^ They have been used across many areas of healthcare including mental health,^17^ e‐health,^19^ osteoarthritis,^16^ interprofessional practice,^20^ and frail older persons' care.^18^ Given the complexity and rapid evolution of CCI, a capability perspective is required as it emphasizes integration of knowledge, skills, values, and attitudes to enable adaptation to change, continuous growth and improvement.^16^, ^21^ Of interest, only one of the aforementioned capability frameworks was developed in consultation with consumers.^16^ Additionally, with the exception of the framework presented in this paper, the research team was not aware of any published health consumer‐focussed capability frameworks.

Given the utility of capability frameworks to guide learning and development and the apparent lack of literature addressing capabilities for successful staff and consumer partnerships in QI, the current research team, inclusive of two consumers, coproduced a scoping review on the topic.^22^ This led to the development of a *Capability framework for successful partnerships in healthcare QI*. The papers in that review originated from nine countries and spanned the healthcare continuum, different health conditions, and a diversity of CCI stakeholders. However, because few papers explicitly discussed capabilities, the framework was based on inferred content. Additionally, the research team members were all from Australia. Hence, the framework needed to be subjected to further scrutiny from a more diverse group to assess its international acceptability and enhance its relevance. This led to the current study which aimed to refine the *Capability framework for successful partnerships in healthcare QI*. Study objectives were: (i) To develop international expert consensus regarding the framework items and structure; and (ii) to explore whether different capabilities are required across diverse healthcare contexts.

## 2 METHODS

**Figure 1:**
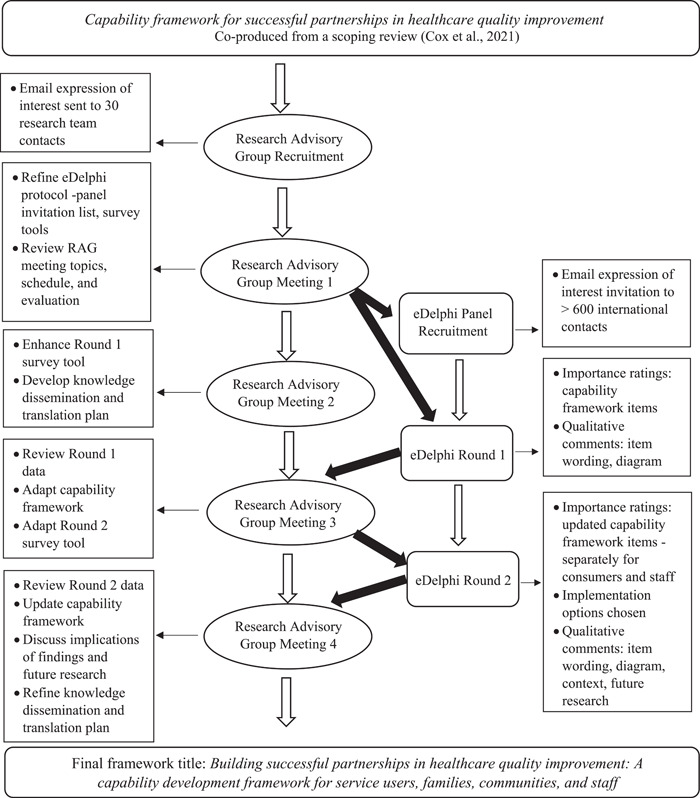
eDelphi study design flow chart

An eDelphi design enabled geographically diverse participants to iteratively contribute to consensus development in an anonymous, convenient, and reflexive manner.^23^, ^24^ Furthermore, an eDelphi study is participatory and inclusive, and explores not just ‘what is’ but ‘what could/should be’.^24^ Although the research team included two consumers, the expertize of a Research Advisory Group, was integral to the study design to maximize the diversity of consumer and other stakeholder perspectives for study decision making and results interpretation. The Research Advisory Group met four times over 4 months. Figure 1 summarizes the study processes including Research Advisory Group meetings and topics, and eDelphi rounds. Recruitment and data collection occurred from June to September 2021. There was no remuneration for any of the consumers involved in this study due to a lack of funding.

### 2.1 Participants and recruitment

#### 2.1.1 Research advisory group

Thirty email invitations were sent to consumers, clinical and nonclinical healthcare staff, and researchers. Study and eligibility information was provided, as was a link to a 7‐minute video describing the justification, development, and content of the capability framework. An expression of interest (EOI) form collected participant background data and was used to screen eligibility. The inclusion criteria were: willingness to participate; high level of English language proficiency; ability to attend videoconference meetings within the study time zone; and ability to demonstrate a high level of expertize in QI partnerships in healthcare through one or more of the following: (i) membership on a healthcare committee(s) which included consumer partnerships for QI for at least two years; and/or (ii) active participation in QI projects which included consumer partnerships or consumer partnering topics for at least two years; and/or (iii) authorship of a peer‐reviewed publication(s) regarding partnerships in healthcare QI; and/or (iv) recognized consumer advisory role regarding QI such as through a consumer‐led organization which supports health services. A Research Advisory Group size of approximately 10–12 people, including the research team, was planned to enable rich discussion. Additionally, the research team aimed for at least half of the Research Advisory Group to have a consumer role.

#### 2.1.2 International expert panel

Consistent with the aims of this study, it was important to recruit International Expert Panel members who had diverse perspectives including people from culturally and linguistically diverse, disability, LGBTIQ+, and indigenous backgrounds. Recruitment was limited to participants from high‐income countries, in keeping with the parameters of the original scoping review.^22^ The invitation list was developed from research team networks and reviewing relevant publication author details. These strategies resulted in limited consumer contacts. Thus, extensive online searches were conducted for health consumer nongovernment organizations and charity email addresses in Australia, New Zealand, the United States of America, the United Kingdom, Canada, Singapore, and Hong Kong. The latter two countries were included as there is a knowledge gap regarding CCI in Asian countries.^25^, ^26^ Over 600 invitations with study background information and the EOI form were emailed. Eligibility criteria were the same as for the Research Advisory Group except that 1 year of experience rather than two was required, and no online meeting availability was needed. Again, the EOI form collected participant background and eligibility information. A panel size of 50 participants was the recruitment goal.

### 2.2 Data collection: eDelphi rounds

Two eDelphi rounds were implemented online (see Figure 1) and were open for three weeks each. Two rounds, rather than three were justified because an initial exploratory round was not required as this eDelphi was based on the previously co‐produced capability framework.^22^ There were approximately three weeks between survey rounds. A systematic review of 100 Delphi studies reported a median consensus level of 75%.^27^ Therefore, this consensus level was adopted.

#### 2.2.1 Round 1

To prepare the panel for involvement, eligible participants were emailed background information and a link to the same explanatory video used with the Research Advisory Group. The round 1 survey asked a small number of demographic questions to assist data analysis while protecting privacy. The panel was requested to rate the importance of each domain and capability description on a four‐point Likert scale from ‘very important’ to ‘not important’. As recommended elsewhere,^28^, ^29^ options for free‐text comments regarding enhancements, plainer language, and suggested additional items were included. The panel was also asked to rate the overall usefulness of the framework in supporting successful healthcare QI partnerships and comment on the summary diagram. Following a request from the Research Advisory Group, the panel was also asked if they wished to rate the framework items separately for consumers and healthcare staff in round 2.

#### 2.2.2 Round 2

A summary of round 1 results, including changes to the capability framework item wording, was emailed to all panel members with the round 2 survey hyperlink. Round 2 included an education level question as requested by the Research Advisory Group. Additionally, the panel was asked to rate their level of agreement that the updated title, new purpose statement and new underpinning principles adequately described framework intent and content. Separate importance ratings of the updated domains and capability descriptions were sought for consumers and healthcare staff, as more than 50% of the panel requested this in round 1. Free text comments and suggestions for additional items were again requested. The panel was also asked to rate the overall usefulness of the updated framework and updated summary diagram. Additionally, they were asked to indicate how they thought the framework could be used to support healthcare QI partnerships. Comments were also sought regarding contextual issues which may limit or promote framework use.

### 2.3 Data analysis

All data were initially analyzed by the first author and then reviewed by the full research team with reference to the raw data. Once an agreement was reached, preliminary results were discussed with the Research Advisory Group. Quantitative data were analyzed descriptively and presented in tables and graphs. Additionally, for round 2, the Kruskal–Wallis *H* test was applied to determine the statistical significance of any differences in ratings of ‘usefulness’ and ‘importance’ for all questions regarding the framework items for (i) participants in Australia compared to those outside Australia (country); and (ii) participants identifying as a consumer and/or carer and nonconsumers (role). Furthermore, the *χ* ^2^ test was used to determine the statistical significance of any differences regarding the choice of potential implementation options by country or role as above. Wilcoxon‐signed ranks were utilized to test whether there was a statistically significant difference in all participants' importance ratings of the capability framework items for consumers versus staff. The *p*‐value was set at .01 for statistical power given there were a large number of comparisons across a relatively small sample size. Statistical analyses were completed using PSPP (Version 1.4.1).^30^ Qualitative descriptive analysis included identification of common positive feedback ideas, suggestions for improvement, challenges to items and queries regarding wording, as recommended elsewhere.^29^

## 3 RESULTS

### 3.1 Participants

Seven people from diverse backgrounds were recruited to the Research Advisory Group resulting in a membership of 12 people (including the research team). The eDelphi panel included 53 people, with 44 (83%) and 42 (77. 8%) participating in rounds 1 and 2, respectively. Table 1 summarizes participant characteristics and indicates that the panel was diverse on multiple characteristics, and that recruitment aims were met. The average age of Research Advisory Group members was 56.0 years (range: 28–74 years). The average age of the round 1 and round 2 panels was 50.9 (range: 27–75 years) and 52.3 years (27–88 years), respectively. Of note, 58.3% of the Research Advisory Group, 65.9% of the round 1, and 65.9% of the round 2 panel indicated a consumer role (some were additional to being a researcher or healthcare staff member). The highest education level was the completion of a Masters degree or above for 58.33% of the Research Advisory Group, and 57.15% of the round 2 panel.

**Table 1:** Characteristics of Research Advisory Group,^a^ and International Expert Panel members in Round 1 and Round 2

Characteristic	Category	Research Advisory Group^a^ (*n* = 12)		International Expert Panel			
				Round 1 (*n* = 44)		Round 2 (*n* = 42)	
		*n*	%	*n*	%	*n*	%
Gender identity	Female	9	75.0%	34	77.3%	30	71.4%
	Gender diverse	–	–	1	2.3%	2	4.8%
	Male	3	25.0%	9	20.5%	10	23.8%
Country	Australia	12	100%	28	63.6%	25	59.5%
	Canada	–	–	5	11.4%	5	11.9%
	New Zealand	–	–	1	2.3%	1	2.4%
	Singapore	–	–	4	9.1%	4	9.5%
	Sweden	–	–	1	2.3%	1	2.4%
	The Netherlands	–	–	1	2.3%	1	2.4%
	UK	–	–	3	6.8%	3	7.1%
	USA	–	–	1	2.3%	2	4.8%
Diversity indicators^b^	Culturally diverse	3	25.0%	8	18.2%	8	19.1%
	Non‐English language	2	16.7%	6	13.6%	6	14.3%
	Living with disability	2	16.7%	6	13.6%	7	16. 7%
	Carer of person with disability	1	8.3%	5	11.4%	6	14.3%
	Older person >65 years	4	33.3%	9	20.5%	10	23.8%
	Carer of older person	2	16.7%	9	20.5%	9	21.4%
	Chronic condition	4	33.3%	14	31.8%	14	33.3%
	Rural or remote	1	8.3%	6	13.6%	6	14.3%
	LGBTIQ+	1	8.3%	3	6.8%	3	7.1%
	Other diversity	–	–	2	4.5%	2	4.8%
	Australian Aboriginal	1	8.3%	1	2.3%	1	2.4%
Role in quality improvement (QI) partnerships^b^	Consumer/patient	3	25.0%	13	29.6%	14	33.3%
	Carer of patient	–	–	10	22.7%	10	23.8%
	Healthcare staff	4	33.3%	19	43.2%	17	40.5%
	Researcher or academic	3	25.0%	22	50.0%	23	54.8%
	Staff or member of consumer organization	2	16.7%	11	25.0%	9	21.4%
	Other	–	–	1	2.3%	1	2.4%
QI in healthcare expertize^b^	Committee member	11	91.7%	34	77.3%	30	71.4%
	QI projects	11	91.7%	42	95.5%	41	97.6%
	Publication	8	66.7%	22	50.0%	24	57.1%
	Consumer organization representative for QI	6	50.0%	26	59.1%	23	54.7%

^a^ Includes research team.

^b^ Participants may have indicated more than one characteristic.

### 3.2 eDelphi Round 1

In round 1, all responses (*n* = 44) were complete, and 95.5% of the panel rated the framework as ‘very useful’ (56.8%) or ‘useful’ (38.6%) in supporting successful partnerships in QI. Table 2 includes the original and proposed wording of domains, capabilities, and descriptions with the percentage of ‘important’ or ‘very important’ ratings. The three capability domains and the *Sharing Power and Leadership* foundational capability were rated as ‘very important’ or ‘important’ by more than 95% of participants. Rewording was suggested for two domains. Of the 27 capability descriptions, one—*(7c) Demonstrates awareness of relevant clinical processes and has sufficient health literacy*, did not reach the 75% threshold. However, the Research Advisory Group advocated for it to be retained and reworded with a health literacy focus for round 2. Rewording was suggested for 21 (77. 8%) of the capability descriptions. There were no consistent suggestions regarding additional capability items. A total of 65.9% of participants wanted to rate the capability items separately for consumers and staff in round 2.

**Table 2:** Original and updated wording of domains, capabilities, and descriptions with ‘importance’ ratings, and statistical significance of rating differences for consumers compared to healthcare staff (round 2 only)

Original wording	Round 1		Round 2			
	% Rated important or very important (*n* = 44)	Proposed rewording after Round 1	% Rated important or very important consumers (*n* = 41)	% Rated important or very important staff (*n* = 39)	Comparison of ratings for consumers versus staff^a^; *p* < .01	Final wording after Round 2
***Personal Attributes Domain***	95.4%	***Personal Attributes Domain***	*92.7%*	*100%*	*ns*	***Personal Attributes Domain***
1. Dedicated to improving healthcare		1. Dedicated to improving healthcare				1. Dedicated to improving healthcare
1a) Motivated to improve patient care and outcomes	97.7%	1a) Motivated to improve person‐centred care and health outcomes	97.6%	100%	*ns*	1a) Motivated to improve person‐centred care and health outcomes
1b) Demonstrates ongoing commitment including sustained participation	84.1%	1b) Demonstrates ongoing commitment	85.4%	97.4%	*p* = .001	1b) Demonstrates meaningful commitment
2. Self‐aware and reflective		2. Being self‐aware and reflective				2. Being self‐aware and reflective
2a) Engages in reflective and reflexive practices that contribute to change for everyone rather than personal interests	97.7%	2a) Engages in reflective and reflexive practices that contribute to equity and achieving positive change	85.4%	97.4%	*p* = .005	2a) Open to engaging in self‐reflection to contribute to achieving positive change
2b) In tune with how one's presence, emotional reactions and behaviours influence others	88.6%	2b) Adapts own behaviour to ensure the inclusion of others	90.2%	94.9%	*ns*	2b) Adapts own behaviour to ensure the inclusion of others
3. Confident and flexible		3. Being flexible and developing confidence				3. Being flexible and developing confidence
3a) Confident to actively engage in constructive dialogue in a group setting including patients, public, healthcare staff, and leaders	79.5%	3a) Builds confidence to actively engage in inclusive, respectful, and meaningful dialogue	95.1%	100%	*ns*	3a) Builds confidence to actively engage in inclusive, respectful, and meaningful dialogue
3b) Flexibly works in unfamiliar and evolving situations	90.9%	3b) Adapts to unfamiliar and evolving situations	90.2%	97.4%	*ns*	3b) Adapts to unfamiliar and evolving situations
***Relationships and Communication Domain***	*100%*	***Relationships and Communication Domain***	*95.1%*	*100%*	*ns*	***Relationships and Communication Domain***
4. Working and learning as a team		4. Working and learning as a team				4. Working and learning as a team
4a) Works as an effective and active member of a team	97.7%	4a) Works as an effective and engaged member of a team	97.6%	100%	*ns*	4a) Works as an effective and engaged member of a team
4b) Recognizes the unique and valuable contributions of each team member	100%	4b) Recognizes the unique and valuable contributions of each team member	92.7%	100%	*p* = *0.008*	4b) Recognizes the unique and valuable contributions of each team member
4c) Embraces colearning	95.5%	4c) Embraces colearning	87.8%	94.9%	*ns*	4c) Embraces learning together
5. Collaborating and communicating		5. Collaborating and communicating				5. Collaborating and communicating
5a) Works collaboratively to build consensus	90.9%	5a) Works collaboratively including presenting an alternative position but respecting group decisions	87.8%	97.4%	*ns*	5a) Works collaboratively including presenting an alternative position but respecting group decisions
5b) Demonstrates strong conflict resolution and negotiation skills	88.6%	5b) Demonstrates strong conflict resolution and negotiation skills	82.9%	97.4%	*p* = .001	5b) Demonstrates conflict resolution and negotiation skills
5c) Builds respectful, constructive, and reciprocal relationships	100%	5c) Builds respectful, constructive, and reciprocal relationships that recognize diverse viewpoints	95.1%	97.4%	*ns*	5c) Builds respectful, constructive, and reciprocal relationships that recognize diverse viewpoints
6. Advocating for everyone		6. Advocating for improvement and equity				6. Advocating for improvement and equity
6a) Influences change to improve patient and public involvement (PPI)	97.7%	6a) Influences change to build and promote partnerships in service improvement	85.4%	100%	*ns*	6a) Influences change to build and promote partnerships in service improvement
6b) Promotes the needs of marginalized populations	90.9%	6b) Prioritizes engagement of populations who experience health inequities	85.4%	97.4%	*ns*	6b) Prioritizes finding ways to engage populations who experience health inequities
6c) Shares successes, networks and links diverse stakeholders	100%	6c) Links with diverse networks to share successes and learnings	92.7%	97.4%	*ns*	6c) Links with diverse networks to share successes and learnings
***Philosophies, Models and Practices Domain***	*95.5%*	***Principles and Practices Domain***	*73.2%*	*100%*	*p* = .001	***Principles and Practices Domain***
7. Organizational systems and policies		7. Influencing organizational systems and policies				7. Influencing organizational systems and policies
7a) Works within organizational priorities, governance, policies, resources, and constraints	75%	7a) Contributes to innovation in implementation and development of organizational priorities, governance, policies, and resources	78.1%	100%	*p* = .003	7a) Contributes to innovation in implementation and development of organizational priorities, governance, policies, and resources
7b) Develops sustainable solutions that fit the context	88.6%	7b) Develops person‐centred, creative, and sustainable solutions	82.9%	100%	*ns*	7b) Contributes to the development of person‐centred, creative, and sustainable solutions
7c) Demonstrates awareness of relevant clinical processes and has sufficient health literacy	72.7%	7c) Seeks to improve health literacy at the individual, organizational, and community levels	89.5%^b^	97.3%^c^	*p* = .003	7c) Seeks to improve health literacy at the individual, organizational, and community levels
8. PPI best practice		8. Implementing partnership best practices				8. Implementing partnership best practices
8a) Committed to the inherent value of PPI	100%	8a) Committed to the inherent value of partnerships	97.6%	100%	*ns*	8a) Commits to the inherent value of partnerships
8b) Implements a variety of PPI principles and practices	90.9%	8b) Tailors partnership approaches to the needs of those involved and the improvement context	75.6%	100%	*p* < .001	8b) Tailors partnership approaches to the needs of those involved and the improvement context
8c) Effectively conveys own experiences to influence and persuade	88.6%	8c) Effectively conveys own experiences to enable positive change	95.1%	97.4%	*ns*	8c) Appropriately conveys own experiences to enable positive change
8d) Facilitates teaching and learning including mentoring/coaching	95.5%	8d) Facilitates teaching and learning including mentoring/coaching	78.1%	97.4%	*p* = 0.002	8d) Facilitates teaching and learning including mentoring/coaching
8e) Provides ongoing support and feedback to patient partners	93.2%	8e) Provides ongoing support and feedback to all partners	78.1%	100%	*p* = 0.001	8e) Contributes to providing ongoing support and feedback to all partners
9. QI principles and processes		9. Using QI principles and processes				9. Using QI principles and processes
9a) Implements appropriate QI processes across service planning, design, delivery, and evaluation	93.2%	9a) Implements contemporary QI processes across service planning, design, delivery, and/or evaluation	70.7%	100%	*p* < .001	9a) Contributes to implementation of contemporary QI processes across service planning, design, delivery, and/or evaluation
***Sharing Power and Leadership—Across All Domains***	97.7%	***Equalizing Decision Making, Power, and Leadership—Across All Domains***	*80.5%*	*100%*	*p* = .002	***Equalizing Decision Making, Power, and Leadership—Across All Domains***
10a) Contributes to transforming traditional power dynamics	97.7%	10a) Continuously works to equalize power differences	75.6%	97.4%	*p* = .001	10a) Commits to equalizing power differences
10b) Actively encourages shared decision making	100%	10b) Engages in shared and inclusive decision making	97.6%	100%	*p* = .001	10b) Engages in shared and inclusive decision making
10c) Supports patient‐led leadership models	97.7%	10c) Supports service user leadership development initiatives	78.1%	97.4%	*ns*	10c) Supports service user leadership development initiatives

^a^ Significant difference of importance ratings for consumers versus staff, based on Wilcoxon‐signed ranks test, *p* < .01.

^b^ *n* = 39.

^c^ *n* = 37.

#### 3.2.1 Suggestions for rewording and additions to the framework

A particular focus in the suggestions for rewording was the inclusivity of diverse consumer stakeholders. Other rewording suggestions were directed at ensuring that QI innovation and change were not inadvertently stifled:

PPI is not about consensus!! It may be the opposite; it may be about exploring conflicts (healthcare staff member, researcher/academic).

Sometimes QI within organizations has to push against entrenched policies, procedures etc. (patient, researcher/academic, staff or member of consumer organization).

Rewording was recommended for the foundation capability of *Sharing Power and Leadership* to focus on equalizing decision making, power and consumer leadership:

Developing leadership capability among consumers is a key aim so that organizations can achieve ‘nothing about us, without us’ (carer, healthcare staff member, researcher/academic).

The word *building* was suggested as an addition to the title, consistent with the house imagery of the framework summary diagram. There were also several requests for a new section at the beginning of the framework to clarify intent and scope with the strong suggestion that growth, development, respect, accountability, and trust be explicitly stated. An emphasis on diversity and the impact of colonization on First Nations peoples was also requested. The Research Advisory Group supported the inclusion of these concepts in the principles. The house diagram was endorsed due to the metaphor of strong foundations in power‐sharing. It was suggested that ‘knowledge, skills and attitudes’ be removed to simplify the image and for colours be added to focus the eye on the vertical capability domains.

### 3.3 eDelphi Round 2

Of the 42 Round 2 responses, five were incomplete. All available data were included in the analyses. The number of respondents for each question is stated below. All participants (100%, *n* = 39) rated the framework as ‘very useful’ (84.6%) or ‘somewhat useful’ (15.4%) in supporting successful partnerships in QI. Table 2 includes the original and proposed wording of domains, capabilities and descriptions with percentage of ‘important’ or ‘very important’ ratings and statistical significance of rating differences for consumers compared to staff in Round 2. There were many comments that the revised wording had addressed Round 1 concerns and had added clarity. In addition, the iterative eDelphi methodology was recognized by participants as welcoming and encompassing consumer perspectives. There was also positive feedback about the emphasis on development:

From my experience, people are looking for tools to help them work in partnership… The capabilities give useful guidance… it's important to communicate that some of the capabilities will be developed through their [consumer] participation (carer).

#### 3.3.1 Title, purpose statement, principles and diagram

**Figure 2:**
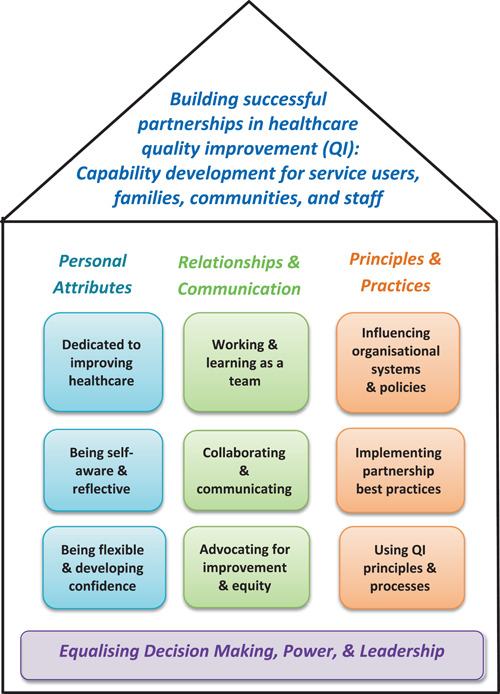
Final capability development framework summary diagram

There was consensus that the updated title adequately described the intent and contents of the framework with 97.6% of participants indicating ‘strongly agree’ or ‘agree’ (*n* = 42). Some concern was expressed that including all iterations of patient/consumer made the title too long. However, for inclusiveness, the Research Advisory Group agreed to include a comprehensive list. The new purpose and principles statements were strongly supported with 100% and 92.9% of panel members indicating ‘strongly agree’ or ‘agree’, respectively, that they described the intent and contents of the framework (*n* = 42). Some concerns were expressed that the statements were too detailed and lengthy. However, this was contrasted with proposals suggesting additional content. Table 3 includes the final reworded framework title, purpose statement and principles. The updated colour summary diagram was supported by 100% (*n* = 39) of participants as ‘very useful’ or ‘somewhat useful’ with comments that it had been improved (see Figure 2).

**Table 3:** Final capability development framework title, purpose statement, and principles

**Title**: *Building successful partnerships in healthcare quality improvement: A capability development framework for service users, families, communities, and staff*
**Purpose**:
1. To describe the key capabilities needed for building successful partnerships in healthcare quality improvement; and 2. To promote reflection, growth, learning and development regarding these capabilities at individual, team, and organizational levels.
**Principles**:
1. Everyone is on a learning journey and this framework intends to support life‐long learning and development for all partners. It is not intended to imply that all partners will begin with all capabilities. 2. Successful partnerships happen in organizational and social contexts, and it is essential that everyone feels welcome, empowered, responsible, trusted, and accountable. 3. Capabilities include knowledge, skills, attitudes, and values which influence behaviour and go beyond competence to include a focus on personal growth and adaptation to change. 4. Organizational leaders have a key role in fostering, resourcing, and promoting a supportive, respectful culture for successful partnerships. 5. Partnerships must occur with diverse individuals and communities across the lifespan including Australian Aboriginal and Torres Strait Islander peoples, and other indigenous peoples internationally; people with a disability; people who identify as LGBTIQ+; people from culturally and linguistically diverse backgrounds; people from rural and remote areas; and all people who experience health inequities. 6. Knowledge and understanding of the history of colonization and the current impact on indigenous peoples lays a foundation for moving forward. 7. Service users, patients, consumers, citizens, family members, carers, friends, community, clinical, and nonclinical health service staff and consumer organization staff, volunteers and consumer advisors are all a focus for this framework. It is also inclusive of current, past or potential users of health services. 8. There is no ‘one size fits all’ method of successful engagement. Appropriate strategies will depend on many factors including improvement goals and available resources.

#### 3.3.2 Separate importance ratings for staff and consumers

The capability domains and all capability descriptions were strongly supported as ‘very important’ or ‘important’ for healthcare staff due to their organizational knowledge and power (see Table 2). For consumers, the *Principles and Practices Domain* did not meet the 75% agreement threshold (73.1% ‘very important’ or ‘important’; *n* = 41). However, within that domain, only one capability description out of the nine did not meet 75% agreement: (*9a*) *Implements contemporary QI processes across service planning, design, delivery, and/or evaluation* (70.7% ‘very important’ or ‘important’; *n* = 41). This suggested that the domain should remain for consumers. This was confirmed by the Research Advisory Group. All other capability descriptions met the inclusion threshold for consumers. There were no consistent recommendations regarding suggested new capability items. Statistically significant differences in importance ratings of capability framework items for consumers versus staff were found for the *Principles and Practices* domain, the *Sharing Power and Leadership* foundational capability and for 12 capability descriptions (44.4%) with all being rated as less important for consumers (see Table 2).

Qualitative data also highlighted panel perceptions that staff and consumer responsibilities were different for capability development. Participants emphasized the duty of paid staff, who are in positions of privilege and power, to value authentic consumer engagement by developing their own capabilities and supporting consumers. Concern was expressed that consumers should not be ‘held to extra standards’ or disadvantaged by the framework and so staff should do the ‘heavy lifting’ (researcher/academic). Several panel members also indicated that some capabilities were more relevant for staff facilitating the QI group/initiative and/or for paid patient advisors/peer workers. Similar to Round 1, and strongly supported by the Research Advisory Group, comments highlighted that power‐sharing and a supportive organizational environment are vital to foster consumer capability development:

While consumers need to understand what's possible in terms of best practices and aspire to this, they are usually much more ready and willing to be at the table, than services providers are to truly welcome them there. Consumers can only lean in if health professionals lean out (staff or member of consumer organization).

Several panel members commented on the importance of consumer remuneration to level the playing field and this was reinforced by the Research Advisory Group. The panel also warned that frameworks and toolkits cannot ‘… magically reduce the volatility, uncertainty, complexity, and ambiguity that is engagement’ (staff or member of consumer organization). Several comments emphasized the aspirational nature of the framework, and that guidance would be required regarding how to develop capabilities.

#### 3.3.3 Comparison of ratings by country and by role

There was no statistically significant difference in ratings of ‘usefulness’ and ‘importance’ for all questions regarding the framework items by country or role. This result suggested that Australians rated the framework items similarly to participants from other countries and that consumers and carers rated the framework items similarly to other panel members.

#### 3.3.4 Implementation strategies

The most frequently endorsed strategies for implementing the framework were: QI team or committee (inclusive of consumers and healthcare staff) reflection and development planning (92.3%, *n* = 39); and, individual reflection and development planning for healthcare staff (alone or with a supervisor or mentor) (84.6%, *n* = 39) (see Table 4). Other implementation suggestions included: using the framework to guide a community of practice; aligning QI activity streams with capability domains for learning events and planning fora; and government system‐level review of QI policies and practice. The current lack of formal structures in most organizations to promote individual consumer development planning was noted. Several panel members suggested that the framework should be validated for research partnerships.

**Table 4:** Strategies for implementation of the capability development framework

Strategies for implementation	% Respondents agreeing (*n* = 39)	*n*
Quality improvement (QI) team or committee (inclusive of consumers and healthcare staff) reflection and development planning	92.3%	36
Individual reflection and development planning for healthcare staff (alone or with a supervisor or mentor)	84.6%	33
Healthcare organization review and gap analysis of resources/materials for planning capability development initiatives, for example training, mentoring, communities of practice	82.1%	32
Healthcare organization review for the development of QI role statements or selection criteria for healthcare staff	76.9%	30
Healthcare organization review for the development of QI role statements or selection criteria for consumers	76.9%	30
Consumer organization review and gap analysis of resources/materials for planning capability development initiatives, for example, training, mentoring, communities of practice	74.4%	29
Individual reflection and development planning for consumers (alone or with a supervisor or mentor)	71.8%	28
Healthcare organization staff training needs analysis questionnaire development	71.8%	28
Consumer organization staff or consumer representative training needs analysis questionnaire development	71.8%	28
Consumer organization review for the development of QI role statements or selection criteria for consumer representatives	71.8%	28
Consumer organization review for the development of QI role statements or selection criteria for consumer organization staff	69.2%	27
Other, for example, using the framework to guide a community of practice	30.8%	12

There was no statistically significant difference regarding the choice of potential framework implementation options by country or role. Many panel members reported that the framework could be universally applied across service delivery models and locations, with the implementation approach, rather than content requiring contextualization. There were comments regarding the influence of organizational factors, such as partnership culture, size and resources, on implementation. General practice, care homes, and small nongovernment organizations were identified as organizations where implementation may be challenging without support. The profit‐driven US health system and the predominance of top–down approaches to healthcare QI in Singapore were noted as potential country‐specific factors.

## 4 DISCUSSION

This eDelphi study advances CCI practice by refining a capability framework for successful partnerships in QI which was based on a co‐produced scoping review.^22^ International consensus has been established on the content and structure of the framework which is now entitled: *Building successful partnerships in healthcare QI: A capability development framework for service users, families, communities, and staff*. The International Expert Panel and Research Advisory Group, who were highly experienced in CCI for QI, endorsed the framework and its summary ‘house’ diagram as valuable resources to support the growth of authentic and meaningful CCI in QI across healthcare contexts, conditions, and countries. A study strength was the diversity of the panel and Research Advisory Group. Additionally, more than 50% of participants identified as having a consumer role, and the panel was drawn from eight countries. While the overall structure of the original capability framework did not change, participant perspectives resulted in substantial rewording and a stronger emphasis on growth, trust, respect, inclusivity, diversity, and challenging the status quo. Capability frameworks have been recommended as beneficial tools to promote individual learning and development, and for organizational priority setting regarding staff development.^16^, ^17^, ^19^ Thus, this study provides direction for the planning and provision of learning and development in QI partnership capabilities which have been identified as important to enhance meaningful CCI.^8^, ^9^, ^15^, ^24^, ^31^ To the research team's knowledge, this is the first capability framework published in the healthcare literature that focuses on both consumers and staff.

### 4.1 Capability development framework content and structure

The capability development framework includes three capability domains: *Personal Attributes*; *Relationships and Communication*; and *Principles and Practices*. In addition, the *Equalizing Decision Making, Power, and Leadership* capability is positioned across all domains and is located in the summary diagram as the foundation of the framework. Ten capabilities with 27 descriptions that define their content and incorporate knowledge, skills, attitudes, values, and behaviours are included. Hence, the framework is consistent with literature that has identified that effective CCI is predicated on values and relationships,^32^ positive attitudes, and behaviours.^31^ The enhanced emphasis on trusting relationships, colearning, and valuing different perspectives as key mechanisms for success, is compatible with coproduction research,^33^ and literature regarding CCI with diverse and under‐engaged populations.^9^, ^34^ It also reinforces that in addition to their lived experience of healthcare, consumers bring much knowledge, skill, and experience to CCI activities.^35^, ^36^, ^37^ The addition of the purpose and principles statements further increases the prominence of CCI core values.

Originally the framework included a capability regarding commitment to sustained participation. ‘Meaningful participation’ was recommended as more appropriate to acknowledge the challenges many consumers face due to personal circumstances. The framework includes staff capability to embed flexibility for consumer schedules, health conditions, caregiving, and other responsibilities as discussed elsewhere.^9^, ^24^ As recommended,^1^, ^31^, ^32^ the framework also highlights the importance of growing capabilities for networking, celebrating successes, and sharing learnings to inform future partnerships. The key contribution of consumers in facilitating vital external connections between communities and health services^35^ is also consistent with the framework. Additionally, it supports amplifying the voices of consumers through capacity building to cochair and lead partnerships.^12^, ^32^, ^38^ This also concurs with a call for health professionals to be allies who support and facilitate consumer leadership rather than speaking on consumers' behalf, or directing how consumers engage.^39^ Similarly, effective CCI is reliant on the capability to create a safe environment where there is motivation to recognize and mitigate power imbalances so that decision making is collaborative.^6^, ^8^, ^40^ Key feedback from the panel and reflected in the refined framework, is that all partners need to respectfully embrace differences of opinion to enable innovation and positive change. This aligns with the importance of ‘challenging, respectful and robust debates’ in effective codesign.^40^

### 4.2 Perceived key responsibility of healthcare staff and organizational leaders

An important finding from this study was that, despite all study materials emphasizing that this was an aspirational framework, the eDelphi panel rated the *Principles and Practices* domain, the *Equalizing Decision Making, Power, and Leadership* capability and almost half of the capability descriptions as more important for staff than consumers. This finding conflicted with the research team's philosophical stance that in authentic partnerships all capabilities are equally important for consumers and staff so that power inequities are addressed. However, the results represent the lived experience of both healthcare staff and consumers who are experienced in QI partnerships and reflect literature that highlights the tokenistic nature of many CCI efforts in QI.^8^, ^41^, ^42^ Despite the differences in importance ratings, overall the study indicated that the framework is applicable to both stakeholder groups, as only one capability description (regarding implementation of QI processes and practices), did not meet the threshold for inclusion for consumers. Additionally, statistical analyses indicated that staff and consumer participants rated items in the same way suggesting that both groups recognize that changes are needed to enhance CCI in QI, with staff needing to champion transformation.

Furthermore, participants emphasized the pivotal role of organizational leaders in influencing the success or failure of meaningful CCI, and hence adoption and support of the framework. This is consistent with recommendations that quality projects incorporating CCI need to be aligned with organizational priorities and be sponsored by senior leadership.^31^, ^36^, ^43^ Adequate resources and backing from senior leaders may be essential to avoid unintended negative consequences of codesign such as perpetuating the marginalization of some populations and increased time and costs with no, or limited, outcomes.^32^ Thus, before contemplating the use of the capability development framework, at a minimum, a frank and open discussion regarding partnership culture and resources, and/or use of a tool such as the Measuring Organizational Readiness for Patient Engagement^2^, ^28^ should be implemented. However, the power of ‘bottom‐up’ approaches to shifting organizational culture regarding CCI should not be underestimated.^39^, ^44^ Participants also suggested that some capabilities may be more important for specialist paid consumer facilitator roles. Previous research has explored the relational, communication, professional, and personal capabilities of such positions.^45^ The nuanced applicability of the framework for these roles is a direction for further research.

### 4.3 Context and implementation

The eDelphi panel and Research Advisory Group advised that the capability development framework content may be universal. The context was perceived as influencing implementation strategies rather than framework structure or items. The fact that there was no statistically significant difference in ratings for any aspect of the framework between participants from Australia compared to other countries supports the notion that it may not be country‐specific. However, this requires further investigation as, for example, culturally specific strategies for CCI in research in Asia have been discussed.^25^ Smaller organizations were identified as potentially less able to implement the framework. However, this conflicts with research that identified that smaller, nonteaching hospitals displayed a higher organizational capacity for CCI than some large teaching hospitals pointing to the critical influence of organizational culture and leadership.^2^ Nongovernment organizations, primary care, and residential care were also identified as settings where framework implementation may be challenging. This requires research as there are many examples of highly effective CCI for QI in primary care, in particular.^46^, ^47^ Consumer renumeration was highlighted by some panel members and the Research Advisory Group as critical to successful implementation and is supported by some literature.^12^, ^32^ However, nonmonetary recognition may be just as important.^24^, ^38^

Utilization of the capability development framework for the whole QI team or committee reflection and development planning was the most frequently endorsed implementation strategy. Initiation of a community of practice guided by the framework had traction with the Research Advisory Group. Both of these activities would reinforce the colearning capability which may create a common language, enhance relationships, and reduce power differentials.^5^, ^36^ The capability for staff and consumer self‐reflection is also incorporated in these initiatives which would further reinforce the framework.

### 4.4 Limitations

The average age of participants was 51.11 years, more than 70% identified as female, all had a high level of English language proficiency, and over 55% had a Masters's degree or higher, which may limit study generalizability. However, as called for previously,^9^, ^34^ the eDelphi panel and Research Advisory Group included people with diverse backgrounds which assisted in gaining multiple perspectives. Further research regarding applicably to indigenous cultures is required as only two people with an Australian Aboriginal heritage were included, one in the Research Advisory Group and one on the panel. Furthermore, no people under 27 years of age participated which was an issue given that empowering youth perspectives in QI is essential.^38^ Additionally, only participants from high‐income countries were involved. Of interest, World Health Organization guidelines regarding CCI in healthcare QI do not specify different strategies according to country income,^1^ making this worthy of further research.

## 5 CONCLUSIONS

International consensus has been established on the content and structure of a capability development framework that focuses on building successful staff and consumer partnerships in healthcare QI. The final capability development framework included three domains: *Personal Attributes*, *Relationships and Communication* and *Principles and Practices*. The *Equalizing Decision Making, Power, and Leadership* capability was foundational and positioned across all domains. The Research Advisory Group and eDelphi panel highlighted the pivotal role that staff, and organizational leaders play in promoting and supporting CCI capability development both for staff and consumers. QI teams or committees could use the framework to reflect on their capabilities to inform learning and development. The framework could also guide communities of practice that are inclusive of consumers and staff.

## AUTHOR CONTRIBUTIONS

Ruth Cox led the study as part of her doctoral research which was supervised by Matthew Molineux and Melissa Kendall. All authors contributed to all aspects of the study across the research cycle. Melissa Kendall performed the statistical analyses. Ruth Cox developed the first draft of the manuscript. All authors reviewed and advised Ruth Cox regarding drafts and approved the final manuscript.

## CONFLICTS OF INTEREST

The authors declare no conflicts of interest.

## ETHICS STATEMENT

Ethical approval was granted by the Metro South Health Human Research Ethics Committee (MS HREC/2019/QMS/52675) and the Griffith University Human Research Ethics Committee (GU Ref No: 2019/659).

## Data Availability

Due to confidentiality and the nature of the consent obtained, the raw data cannot be shared. Summary data which support the findings of this study are available from the corresponding author upon reasonable request.
